# Simplified Transformation of *Ostreococcus tauri* Using Polyethylene Glycol

**DOI:** 10.3390/genes10050399

**Published:** 2019-05-26

**Authors:** Frédéric Sanchez, Solène Geffroy, Manon Norest, Sheree Yau, Hervé Moreau, Nigel Grimsley

**Affiliations:** 1 CNRS UMR7232 BIOM (Biologie Intégrative des Organismes Marin) Sorbonne University, 66650 Banyuls sur Mer, France; sanchezf@obs-banyuls.fr (F.S.); manorest@laposte.net (M.N.); sheeyau@gmail.com (S.Y.); herve.moreau@obs-banyuls.fr (H.M.); 2 IFREMER, Centre Atlantique, 44331 Nantes CEDEX 03, France; solene.geffroy@ifremer.fr

**Keywords:** Chlorophyta, prasinophyte, Mamiellophyceae, *Ostreococcus*, plankton, picoeukaryote, ecosystem, bioluminescence, promoter, luciferase, gene expression

## Abstract

*Ostreococcus* *tauri* is an easily cultured representative of unicellular algae (class Mamiellophyceae) that abound in oceans worldwide. Eight complete 13–22 Mb genomes of phylogenetically divergent species within this class are available, and their DNA sequences are nearly always present in metagenomic data produced from marine samples. Here we describe a simplified and robust transformation protocol for the smallest of these algae (*O. tauri*). Polyethylene glycol (PEG) treatment was much more efficient than the previously described electroporation protocol. Short (2 min or less) incubation times in PEG gave >10^4^ transformants per microgram DNA. The time of cell recovery after transformation could be reduced to a few hours, permitting the experiment to be done in a day rather than overnight as used in previous protocols. DNA was randomly inserted in the *O. tauri* genome. In our hands PEG was 20–40-fold more efficient than electroporation for the transformation of *O. tauri*, and this improvement will facilitate mutagenesis of all of the dispensable genes present in the tiny *O. tauri* genome.

## 1. Introduction

Marine microalgae in the class Mamiellophyceae are emerging as useful models for integrative biology for several reasons. They can be easily cultured in the laboratory, witnessed by more than 300 strains maintained in the Roscoff Culture Collection (RCC) in France [1], and they diverged at the base of the green lineage that includes land plants, thus providing a starting point for phylogenetic analyses [2]. Several of their streamlined haploid genomes have been completely sequenced and analysed [3,4,5,6,7,8], and they harbour a minimal number of genes for cellular processes, as reflected in the size of their nuclear genomes. Species in the genus *Ostreococcus,* for example, which so far includes four phylogenetically divergent [9] species with fully sequenced genomes (see [3,4,8] and https://genome.jgi.doe.gov/OstRCC809_2/OstRCC809_2.home.html), have only 12–14 Mb of nuclear DNA, comparable to that of yeast (12.1 Mb) [10]. This is much smaller than those of other model species in the green lineage such as mosses (*Physcomitrella patens*, 476 Mb, [11]) *Arabidopsis thaliana* (135 Mb [12]) or maize (2.4 Gb [13]). *Ostreococcus* spp. thus often possess only one copy of genes that are multigene families in other photosynthetic organisms, so alteration of individual genes by directed mutagenesis immediately gives rise to new phenotypes [14,15]. Various kinds of biological analyses involving these algae are being undertaken, for example, the functional analysis of casein kinase 1 in cell cycle [16], nitrogen status sensing by calcium-dependent protein kinases (CDPKs) [17], adaptation to iron limitation [18] and the diurnal regulation of trehalose [19]. In these cases, genes present in only one or two copies in *O. tauri* may be present as multiple orthologues in other species, for example, there is only one copy of the dynamin central domain in *O. tauri*, but there are 6 copies of it in *C. rheinhardtii* [20]. 

Due to their extremely small size (0.8 to ~2 µm) genera in the order Mamiellales (class Mamiellophyceae) are almost always well-represented in microbial fractions of marine samples collected for metagenomic analyses. They are distributed worldwide in marine environments, their different species dispersed according to trophic conditions and temperatures [21,22,23], being particularly abundant in coastal waters (e.g., [24,25,26]). Given the number of laboratories exploiting these models and the nature of some of the work being undertaken, reliable transformation techniques for these species are indispensable. Until now, the transformation procedure developed has involved electroporation [27]—a procedure used for several other algal systems [28,29]—and this technique has since been used to for transformation in various physiological studies of *O. tauri* (see [30,31,32,33] for further examples). Briefly, the electroporation procedure, which has been explained in more detail in a total of 17 steps [34], involves the preparation of algal material, electroporation, and inclusion in soft agar for plating, and requires two days to complete. We report here on a transformation using polyethylene glycol (PEG) that can be completed comfortably in one day. PEG has previously been used frequently for transformation or transient expression via plant protoplasts (reviewed in [35,36]), and for certain unicellular algal species [37,38,39]. The mechanism by which PEG acts is not very clear, although it is known to act as a molecular crowding agent, increasing the effective concentrations of solutes [40,41], and may promote DNA binding to the cell membrane followed by endocytosis [42,43,44]. Additionally, we developed a cloning and expression vector specifically for this group of prasinophyte algae and tested its function *in vivo.* We began by testing a protocol that was optimized for the single-celled microalga *Cyanidioschyzon merolae* [45], since this organism has a similar size to that of *O. tauri*, and neither of these species have a cell wall discernible by transmission electron microscopy.

## 2. Materials and Methods 

### 2.1. Algal Culture

*Ostreococcus tauri* RCC4221 (Roscoff Culture Collection) was cultured in liquid L1 medium (NCMA, Bigelow Laboratory for Ocean Sciences, USA) prepared using either autoclaved offshore seawater (surface water collected at the MOLA station: 42°27′11″ N, 3°8′42″ E) diluted 10% with MilliQ water to obtain a salinity of about 30 g·L^−1^ (30‰) and filtered prior to use through 0.22 μm filters, or artificial seawater (salinity 30 g·L^−1^ (30‰) https://www.mbari.org/wp-content/uploads/2015/11/KASW.pdf [6,46]). All cultures were maintained under a 12:12 h light:dark regime in 50 μmol photon m^−2^ s^−1^ white light at 20 °C. For maintenance, algal cultures were sub-cultured at a dilution of 1/100 in fresh L1 medium every 7 days. Bacterial contamination was reduced by adding antibiotics (final concentrations 50 µg·mL^−1^ ampicillin, 10 µg·mL^−1^ gentamicin, 20 µg·mL^−1^ kanamycin, and 100 µg·mL^−1^ neomycin, from a 1000× concentrated filter-sterilized stock solution kept at −20 °C). Algal cultures were typically treated with antibiotics about once every 3 months, for one subculturing cycle.

### 2.2. Flow Cytometry

Cells were fixed using glutaraldehyde (0.25% final concentration, for 20 min in the dark). Enumeration of cells was performed using a Beckman–Coulter Cytoflex flow cytometer, by chlorophyll autofluorescence for algae [47] and by addition of SYBR Green I (Ozyme ref LON50512) for bacteria [48]. The same excitation wavelength (laser 488 nm) was used for the detection of chlorophyll (detection filter > 620 nm) and SYBR Green (detection bandwidth 525–540 nm, the FITC (fluorescein isothiocyanate) channel).

### 2.3. DNA Preparation

Plasmid DNA was extracted from bacterial cells using the NucleoBond^®^ PC 500 kit (Machery-Nagel). For each transformation, up to 10 μg of gel-purified linearized plasmid DNA was prepared as follows: (1) Enzymatic digestion by Bgll (Promega R6071, Madison, WI, USA) for 4 h at 37 °C. (2) Preparative electrophoresis to separate the plasmid replicon from the insert. The insert contained an antibiotic resistance gene and a reporter gene, both with appropriate promoter and terminator sequences. The gel was prepared using 0.8% agarose (Euromedex ref. D5-E, Stasbourg, France) with 0.5× TAE buffer [49] and crystal violet at 2 μg·mL^−1^ (Sigma-Aldrich ref.C0775, Saint-Louis, MI, USA). The digested DNA was mixed with loading dye (30% glycerol, 20 mM EDTA, 100 μg·mL^−1^ crystal violet). (3) After migration, we excised the appropriate band and used the Wizard^®^ SV Gel and PCR Clean-Up System (Promega A9281). (4) Just before transformation we mixed each lot of 2–10 μg purified DNA with 1 µL of the yeast tRNA at 2 mg·mL^−1^ (Sigma-Aldrich ref. 10109495001).

### 2.4. Transformation

Polyethylene glycol, PEG MW 4000, 6000 or 8000 (reference 81240 Sigma-Aldrich) solution was prepared by diluting in MilliQ water to obtain a concentration of 60% (*w/v*) before filter-sterilisation (0.22 μm pore size). For each transformation, 50 mL of cells at density of 20–30 × 10^6^ mL^−1^ were used. In our culture conditions this cell density occurred during the late part of the exponential growth, about one week after subculturing.

We then centrifuged the culture for 10 min at 6000× *g* at 20 °C and the pellet was resuspended in a microtube (1.5 mL) with 500 μL of L1 medium to reach a final cell concentration of about 10^9^ cells·mL^−1^. DNA/tRNA was added, then gently mixed in 500 µL PEG (60%), giving a final PEG concentration of 30% (*w/v*), and incubated on the laboratory bench (~10 μE m^−2^ s^−1^) at 20 °C for 2 min. Cells were then diluted into 40 mL of fresh L1 medium, thereby diluting the concentration of PEG to 1.5% in the recovery culture, and transferred to a growth chamber for 2–6 h before plating out.

Transformation by electroporation was done as previously described [27,34]. 

### 2.5. Selection on Plates in Semi-Solid Medium

One millilitre aliquots of a solution of 2.1% (*w/v*) low-melting-point agarose in MilliQ water were maintained at 60 °C in a water bath. For each plate (Petri dish, diameter 55 mm), 8 mL of L1 medium with the required concentration of antibiotic (G418 disulphate salt (Sigma-Aldrich, A1720) at a final concentration of 2 mg·mL^−1^) were added to the tube containing the warm agarose, and quickly mixed. One millilitre of freshly transformed cells in L1 + G418 were then added and poured. Plates were dried by leaving the lids halfway open in a sterile laminar air flow cabinet for 20 min before returning them to the culture chamber (100% humidity) at 20 °C.

### 2.6. Luciferase Assays

We prepared a suspension of algae at 2 × 10^7^ cells⋅mL^−1^, adding aliquots of 200 μL to each well of a 96-well white plate (Greiner Bio-One™ LUMITRAC, SAS, Les Ulis, France), then added D-Luciferin (Pierce, Waltham, MA, USA, ref 88293), final concentration 10–100 μM and placed the plate at 20 °C for 10 min in the dark before measuring luminescence with a Victor3 Multilabel Plate Counter Spectrofluorimeter (Perkin Elmer, Waltham, MA, USA) by digital photon counting.

### 2.7. Pulsed-Field Gel Electrophoresis

Pulsed-field gel electrophoresis (PFGE) and in-gel hybridisation using dried gels [50,51] was conducted as previously described [52,53], with the following modifications. The cell cultures were grown to mid-exponential phase (~1.6 × 10^7^ cells⋅mL^−^^1^), 8.7 × 10^7^ cells were harvested by centrifugation (8000× *g* for 20 min) and resuspended in 150 µL of TE buffer (10 mM Tris-HCl, 125 mM EDTA, pH 8). Cells were embedded into plugs by mixing with an equal volume of molten low-melting-point agarose (1% in TE buffer precooled to 45 °C), to a final volume of 300 µL before adding to moulds (Bio-Rad, Hercules, CA, USA). After setting, cells were then lysed in the plugs using proteinase K buffer (10 mM Tris HCl pH 8, 0.5 M EDTA pH 8, 1% lauryl sarcosinate and 1 mg⋅mL^−1^ proteinase K (final concentration), 37 °C, 24 h under constant agitation), before washing three times in 0.5 M EDTA pH 8 at 37 °C for 2 h under constant agitation and storage at 4 °C. Electrophoresis was performed subsequently in 0.8% agarose gels in 0.5 × TBE buffer (final 44.5 mM Tris, 44.5 mM boric acid, 1 mM EDTA at pH 8) using a CHEF-DR III (Bio-Rad) system. For each sample, 2 mm of plug was loaded into the wells. Electrophoresis was run at 14 °C, 6 V cm^−1^ with 120° pulse angle for 15 h with a switch time of 60 s and followed 9 h at a switch time of 90 s. After PFGE, the gel was moved to flat-bottomed dish and enough 0.4 M NaOH was added to cover the gel for chemical denaturation of the DNA by incubation for 30 min at room temperature with agitation. Then the gel was washed three times for 10 min in a 6 × SSC solution (20 × SSC, 3 M NaCl, 0.3 M Na_3_citrate, pH 7). Finally, the gel was dehydrated under vacuum (Hoefer inc. GD2000, Richmond, CA, USA). 

PCR was used to amplify a 630 bp region of the *G418* gene selectable marker gene with the primer pair Kan-FW CCTGTACGGGTACAAGTGGG and Kan-RV CAGGTGGAACTGGAGCTTGT and the product was cleaned (Wizard^®^, Promega, SV Gel and PCR Clean-Up System). The amplicon was randomly labelled with [α-^32^P]CTP (Perkin-Elmer ref BLU008H250UC) according to the manufacturer’s instructions (Prime-a-Gene kit, Promega ref U1100) for use as a DNA probe. Dried gels were equilibrated in hybridisation buffer (6 × SSC, 5 × Denhardt’s solution, 0.1% (*v*) sodium dodecyl sulphate, 10 μg·mL^−1^ tRNA), radiolabelled probe was added, hybridised overnight at 65 °C, and the gel exposed to radiographic film.

## 3. Results

### 3.1. Choice of Selectable Markers for Transformation

Several antibiotics used successfully in other genetic systems were assessed for their effects on exponentially growing *O. tauri* cells. Nourseothricin and G418 were already known to be effective [27]. We tested ammonium glufosinate [54] (50 μg·mL^−1^ to 2 mg⋅mL^−1^), paromomycin sulphate [55,56] (100 μg·mL^−1^ to 2 mg·mL^−1^) and rapamycin [57] (2–400 μM). None of these were effective.

**Figure 1:**
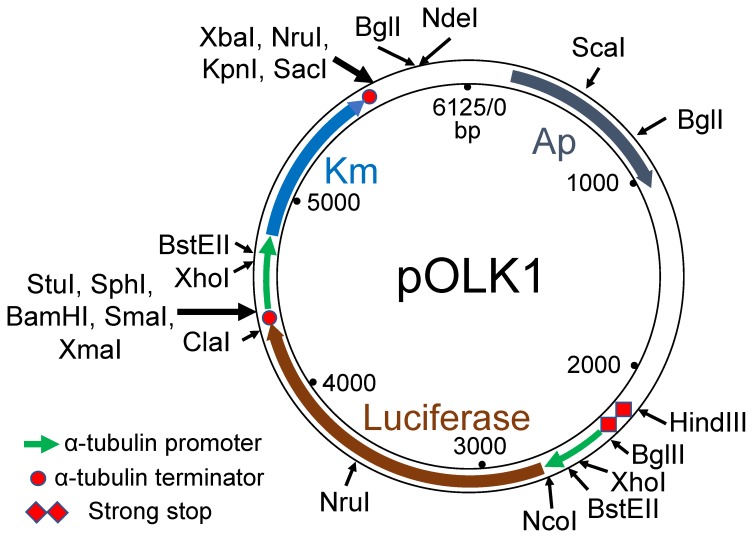
pOLK vectors used for transformation. Km—eukaryotic G418 (or kanamycin) resistance. Synthetic sequences for the visible (Luciferase) and algal selectable (Km) markers were inserted in the pUC57 cloning vector between the HindIII–BamHI sites in the polylinker. Commonly used restriction endonucleases cutting once or twice are shown, and thicker arrows indicate multiple-enzyme cloning sites. In the vectors pOLK2, pOLK3, pOLK4 and pOLK5 (not shown), the BglII–NcoI fragment was replaced by promoter sequences from Histone 2A, Histone 3, Ubiquitin and Thioredoxin, respectively (the sequences of pOLK1 and other component parts are listed in Supplementary File 1, Table S1).

To synthesize a suitable vector, the coding sequences (CDSs) of genetic marker genes were first chosen: (i) the aminoglycoside 3′-phosphotransferase type I (*APH*I) coding gene of the bacterial transposon *Tn903* for G418 resistance [58] and (ii) the firefly luciferase CDS [59] was chosen as a reporter for gene expression. The predicted amino acid sequences of all of these genes were then back-translated using the EMBOSS [60] function *backtranseq* together with a codon usage table for *O. tauri* produced by EMBOSS *cusp*, which was built from the characterised *O. tauri* CDS available from the ORCAE website [61]. These sequences were then edited by replacing codons for glycine, leucine, serine and arginine with the optimum codon sequences for highest expression [62] and, when possible, by introducing a synonymous base substitution in one of the codons in undesirable restriction enzyme sites (SmaI, SacI, XmaI, XhoI from the *APH*I CDS and ScaI, NcoI, KpnI, BglI, BamHI from the luciferase CDS). Next, we chose promoter and terminator regions from highly expressed constitutive genes, firstly α-tubulin because it was shown to have constitutive expression in algae [63,64,65] then histone and thioredoxin genes because they were expressed constitutively in healthy and virus-infected cells [66]. The length of DNA chosen for promoters was rather subjective, in general being about half of the individual intergenic sequences concerned. A “strong stop” (SS1, Figure 1) sequence was used to avoid translational or transcriptional read-through from adjacent sequences. This was generated by using the sequence of a short intergenic region from between two convergently strongly expressed genes (ostta05g02170 and ostta05g02170, *O. tauri* genome version 2 [61]) and adding three out-of-frame translational stops to both ends of the intergenic region. Some commonly used restriction enzyme sites were then added in-between or around the genes to facilitate subsequent cloning steps (Figure 1). The two genes with suitable promoters were then synthesized commercially (Genscript, USA) and ligated to a standard vector pUC57 to produce pOLK1 (Figure 1). Subsequently, three other constructions were produced from the pOLK1 vector by replacing the BglII-NcoI α-tubulin promoter sequence upstream of the luciferase gene with native *O. tauri* promoter sequences for histone 2A (pOLK2), histone 3 (pOLK3), ubiquitin (pOLK4) and thioredoxin (pOLK5).

### 3.2. Optimisation of the Transformation Procedure

**Figure 2:**
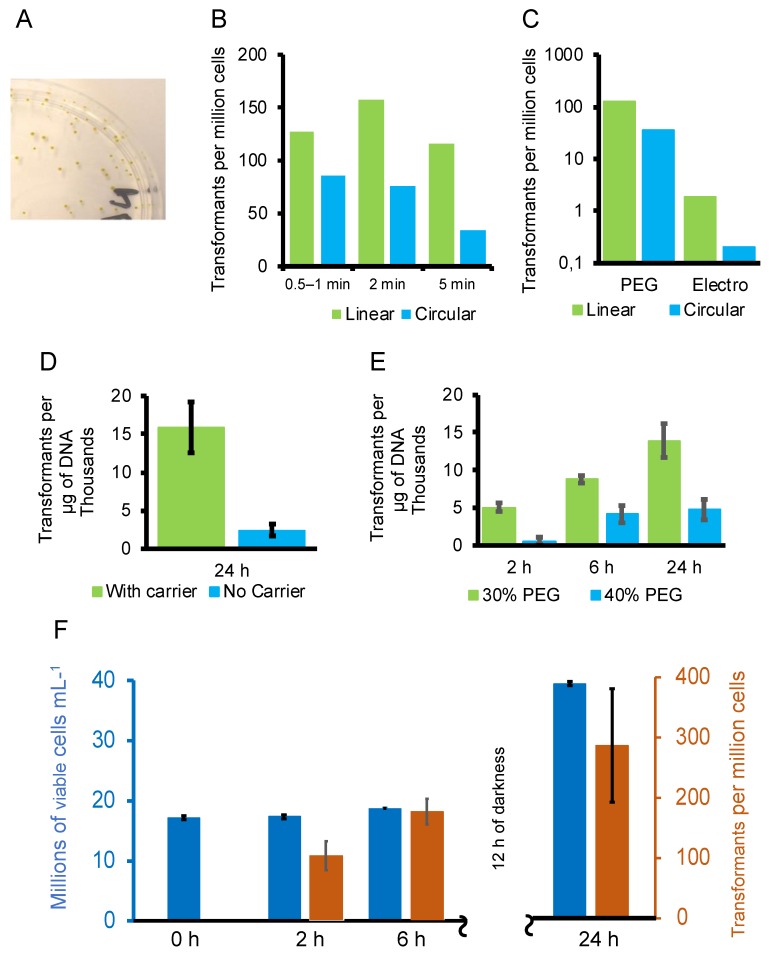
Optimisation of the transformation procedure. (**A**) Colonies of transformants on a Petri dish with 9 cm diameter 19 days after transformation. Linearized DNA gave 2-to-9-fold more transformants than circular DNA in five comparisons (**B**,**C**), 2 min incubation in the PEG + DNA transformation mix was sufficient ((**B**), three comparisons). In (**B**,**C**), recovery of cells in culture after treatment with PEG was 24 h, whereas in (**D**—**F**), the times of recovery of cells in culture after treatment with PEG is shown along the abscissae. PEG was superior to electroporation ((**C**), two comparisons, note the logarithmic scale) and carrier tRNA was advantageous ((**D**), tests in four independent transformations, bars show standard deviations). At lower concentrations of transforming DNA (2 µg) the transformation more often failed completely if there was no carrier (overall 3/11 tests failed with no carrier, data not shown). Either salmon sperm DNA (**B**,**C**) or yeast tRNA (**D**—**F**) were used as a carriers, except for the absence of carrier in one treatment. It was found that 30% PEG MW 4000 was better than 40% PEG MW 4000 ((**E**), three tests). Panel (**F**) shows comparisons of cell cycle stage on the number of transformants on two independent vertical axes. Blue bars (left axis) show cell growth in untreated cell cultures measured at the time of the transformation experiment. Cell density showed only a small increase during the day, but doubled during the night. Brown bars (right axis) show the number of transformants found after three weeks from short (2 min) PEG/DNA treatments and with recovery periods in culture of 2, 6 or 24 h. (**B**) Addition of tRNA carrier increased transformation frequency. (**C**) Increasing the concentration of PEG MW 4000 reduced the yield of transformants. Standard deviations were calculated using the STDEV.S function in Microsoft Excel.

To develop the transformation procedure, we performed a series of test experiments (Figure 2 and Table S2), each conducted over a period of about two months. Usually, four to eight tests were regrouped to be done simultaneously, to allow enough time for handling cells in a day and to make better within-group treatment comparisons. After the PEG treatment, the cells were allowed to recover in culture for 24 h before plating them out unless otherwise stated, to remain consistent with previous studies. The time from transformation to picking off individual colonies on selective plates was usually about 21 days.

In first a series of experiments we compared electroporation [27] with PEG, using PEG conditions similar to those optimised for *C. merolae* [45], and found that in our hands PEG gave about 20–40 times as many transformants than electroporation (Figure 2). We then tested the effects of several other conditions (Figure 2 and Table S2), including the time length of the PEG treatment, the concentration of PEG, the PEG molecular weight, linear or circular transforming DNA, the concentration of transforming DNA, the presence or absence of carrier DNA or tRNA, and the use of different transforming DNAs. Short incubation periods with PEG were found to be optimal (Figure 2), and we retained two minutes as the standard for further tests. 

Flow cytometry measurements confirmed that longer times in the PEG treatment or use of higher-molecular-weight PEG (MW 4000, 6000 and 8000 were tested) were indeed more toxic (Figure S1A,B). Linearized DNA gave 2-to-9-fold more transformants than circular DNA in four comparisons (Figure 2). Increasing the concentration of PEG MW 4000 from 30% to 40% reduced the frequency of transformation (Figure 2). Carrier tRNA increased the transformation frequency (Figure 2D). At lower concentrations of transforming DNA (2 µg) the transformation was more likely to fail completely if there was no carrier (overall 3/11 tests failed with no carrier, data not shown). Overall, comparing between transformations where 2 µg of linear transforming DNA was used, absence of carrier yielded 6.35 × 10^3^ ± 8.44 × 10^3^ transformants per microgram of vector DNA (*n* = 11), whereas salmon sperm carrier DNA gave 1.59 × 10^4^ ± 7.45 × 10^3^ transformants per microgram of vector DNA (*n* = 5) and yeast tRNA carrier yielded 2.11 × 10^4^ ± 4.83 × 10^3^ transformants per microgram of vector DNA (*n* = 4). Since all of the 11 independently transformed lines that we tested by Southern blot carried integrated vector DNA (see Figure 4 in Section 3.3 below), all of these lines were transformed. Negative controls (no vector DNA) were done in all tests (data not shown) but never gave rise to G418-resistant lines. 

In a second series of experiments, the length of time the cells spent recovering in liquid medium in the growth chamber before plating them out on selective medium was tested. In the 12 h light/12 h dark cycle that was used, cell division occurred mainly in the night. Although overnight incubation in culture chambers (up to 24 h) produced about three times as many colonies as 2 h incubation, it is likely that a large proportion of the surviving cells divided once, potentially producing duplicate transformants. The density of cells in control untreated cultures rose only 2% over 2 h (at t = 2 above the t = 0 level, 11:30 a.m.) and 9% at t = 6 h, but at t = 24 h it had risen to 125%, confirming that nearly all cell division occurred in the night (Figure 2 and [67]). In contrast, the number of transformants increased to 177% at t = 6 h (when t = 2 h was arbitrarily considered to be 100%) and to 290% at t = 24 h (Figure 2A). This step was thus shortened to 2–6 h in the optimised protocol presented. Two hours is sufficient to obtain about 20 transformants per million cells input, and 6 h in recovery (before the cells divide) permits about twice this frequency, with only a small risk of finding duplicate transformants if sufficient time is available on the same day to do the experiment.

**Figure 3:**
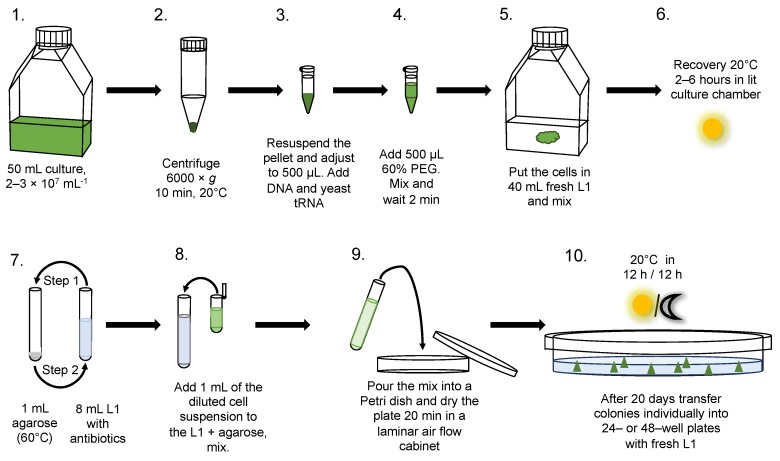
An optimised procedure for transformation of *O. tauri* using polyethylene glycol. **1**, Maintain cultures in a 12 h:12 h light/dark regime, 50 μmol photon m^−2^⋅s^−1^ white light at 20 °C. **2**, Centrifuge 50 mL of the culture at 6000× *g* for 10 min at 20 °C. Discard the supernatant and resuspend the pellet by pipetting in 150 µL L1 medium. Transfer the suspension to a 1.5 mL plastic centrifuge tube. **3**, Adjust the volume to 500 µL with L1. Add 5–10 µg DNA and 1 µL 2 mg·mL^−1^ yeast tRNA. **4**, Add 500 µL PEG (60%), mix and wait 2 min. **5**, Gently pipette the cell suspension into 40 mL fresh L1 medium in a culture flask and mix. Put the flask back to culture for 2–6 h, 50 μmol photon m^−2^ s^−1^ white light at 20 °C. **6**, While the cells are recovering in the 2–6 h incubation, independently prepare 13 mL culture tubes, two for each transformation—one tube containing 1 mL melted agarose held in water bath or incubator at 60 °C, and a second tube containing 8 mL of L1 plus antibiotics kept at 20 °C. **7**, Step 1: Pour the 8 mL of L1 and antibiotics into the tube with 1 mL low-melting agarose at 60 °C. Step 2: transfer all of the mix back to the first tube. **8**, Dilute 200 µL of cells to 1 mL final volume of L1 with antibiotics. Add this diluted cell suspension to the mix of L1 plus agarose and antibiotic solution and mix by inversion. **9**, Pour the mix into a 55 mm diameter Petri dish and dry it for 20 min in a laminar air flow cabinet. **10**, After 20 days, when individual colonies have grown, pick them off in small agarose plugs using plastic micropipette tips and transfer them to 24–48-well plates with fresh L1, culturing each clone in a separate well.

A schematic presentation of this optimised procedure is shown in Figure 3 and described in the Methods section. Correcting for the shorter recovery period in culture of 2–6 h, this procedure gave 10^4^ transformants per microgram of transforming DNA, or about 4 × 10^5^ transformants from each 50 mL flask of cultured cells used in the experiment.

### 3.3. Integration of Transforming DNA is Random

**Figure 4:**
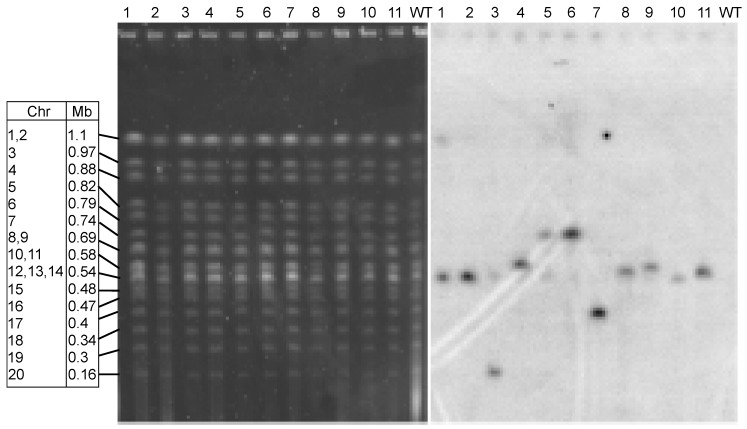
Karyotypes and integration of DNA in transformed lines. Pulsed-field gel electrophoresis (PFGE) separation of chromosomal DNA from *Ostreococcus tauri* lines. **Left panel**: fluorescence after staining the gel with ethidium bromide. **Right panel**: autoradiograph of the same gel probed with the coding sequence of the *APH* gene. WT: wild-type *O. tauri* strain RCC4221 non-transformed control. Numbered gel wells indicate transformed lines, each lane from a single clone. The table on the left displays chromosome number and chromosomal lengths in megabase pairs of the control WT strain. Note: some chromosomes cannot be resolved on this gel.

Radioactive probing of *O. tauri* chromosomes in gels separated by pulsed field gel electrophoresis (PFGE [52] was used to verify that the putative transformants had stably integrated plasmid DNA. The karyotypes of 11 individual colonies arising from transformations using pOLK1 were visualized by PFGE [68]. The gels were then probed with the 0.63 kb long fragment prepared from the *APH*I gene present in the vector. Usually insertions occurred on a single chromosome, visualized as a dark band on the gel track, migrating at the same position as a chromosomal band (Figure 4). 

Insertions appeared to be random, although several occurred on the unresolved groups of chromosomes. In transformants 1, 2, and 10, insertions appeared on the unresolved group of chromosomes 12–14, while in transformants 4, 8, 9, and 11, insertions appeared on the unresolved pair of chromosomes 10 and 11. Other insertions were on well-resolved chromosomes: transformant 3 on chromosome 20, transformants 5 and 6 on chromosome 7 and transformant 7 on chromosome 17.

### 3.4. Transgene Expression from Integrated DNA

In order to test the utility of our vector, and to test several promoters for future work, we replaced the BglII–NcoI fragment of the vector, immediately upstream of the luciferase gene (Figure 1) with four other native *O. tauri* promoter regions (Materials and Methods, and Table S1). Expression from these promoters was assayed in transgenic lines by comparing their bioluminescence. Sixteen independent transformed lines from pOLK1, pOLK2, pOLK3 and pOLK4 were chosen randomly and assayed for expression of luciferase (64 independent transformants in total). All of the independent transformed lines expressed the luciferase reporter gene.

**Figure 5:**
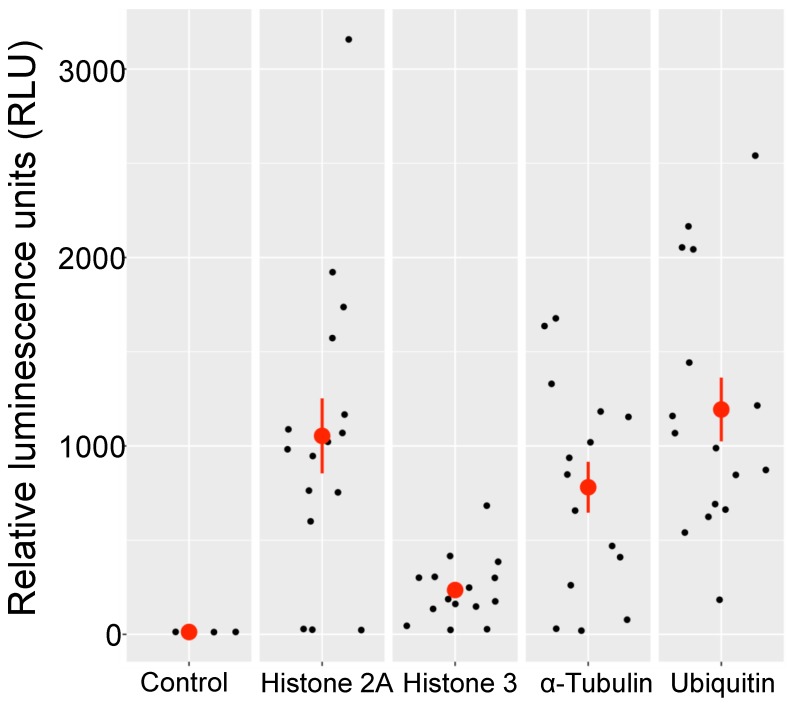
Emission of light from transgenic lines expressing luciferase. Promoters from highly expressed genes were compared (16 independent transformants were tested for each construction): Control: background level of fluorescence from four independently grown non-transformed lines, pOLK2–Histone 2A (P_H2A), pOLK3–Histone 3 (P_H3), pOLK1–α-Tubulin (P_aTub), pOLK4–Ubiquitin (P_Ub). The raw data are available in Table S3. To compare between constructs, we preferred to plot the relative bioluminescence units (ordinate) from each transformant as jittered points [69] (“geom_jitter” ggplot2 function in R). The means and standard errors for each construct are shown as large red dots and vertical bars, respectively.

The majority of these transformants showed high levels of expression, but the level of expression of individual lines was very variable (Figure 5 and Table S3). In relative units, P_Ub and P_H2A gave the highest levels of expression (pOLK2 and pOLK4, overall about one-hundred-fold higher than the control), followed by pOLK4 (P_Ub, 97-fold), pOLK1 (P_aTub, 77-fold) and pOLK3 (P_H3, 23-fold). 

## 4. Discussion

One inconvenience of using electroporation as a means for transformation is that the cells being used must first be washed and resuspended in a non-conductive solution of sufficient osmolarity to prevent them from bursting. Any traces of salt left in the mixture will cause electrical arcing and loss of the cells, so a second washing step is necessary [27]. In contrast, PEG transformation allows cells to be centrifuged and resuspended directly in the same culture medium, and no electroporation step is required.

Once we optimised the use of PEG, we went on to further refine the technique by shortening the delay between transformation treatment and plating out for individual cell lines. Previous protocols [34,37] allowed one day of recovery before the plating out of cells. While this gives rise to more antibiotic-resistant cell lines, an unknown proportion of these lines might arise by cell division, probably allowing duplication of some of the individual transformation events. In control cultures not exposed to PEG, the population density of cells increased in the night (Figure 2 and [67]). Although there was no apparent increase in the number of viable cells after PEG, we do not know whether PEG stopped cell division or whether there was a mixture of some cell division and some cell death. It was not necessary to allow cells to recover overnight, and the cells obtained could be plated out on the same day after the transformation treatment. This modification not only allows completion of the experiment in a single day, but also assures that all of the transformed colonies of cells after the transformation are likely to arise independently. Under the 12 h/12 h day/night growth regime used to grow the cells, the cells normally divide in the early part of the night [67], in contrast to cells grown under continuous illumination [34], whose asynchronous division is about 1.8 per day. This consideration is important for determining the transformation frequency (e.g., when calculating the number of transformations required to mutate all dispensable genes). Reducing the recovery time in culture after PEG treatment to 2 h gave about half as many G418-resistant as at 24 h, and reducing it to 6 h (also possible in one working day) gave about two-thirds the number (Figure S1B). The integration events generally occurred on a single chromosome in each transformed line. According to the variation in band intensity, multiple copies of introduced genes would be integrated into a certain chromosome (Figure 4).

The expression of bioluminescence has been used as a visible reporter successfully in many previous studies in *O. tauri.* As examples, to mention but a few, Corellou et al. [27] used the luciferase expression in transformed lines to investigate circadian gene regulation in an article that also first reported the use of electroporation for *O. tauri* transformation. Luminescence reporter constructs have also been used to investigate the levels of expression of an inducible phosphate transporter [30] and to monitor the involvement of magnesium flux in cellular clocks [70]. We thus chose luciferase as a marker in PEG-mediated transformation based on the strength of its use in previous work. However, while all of these studies used purpose-made promoter-luciferase constructions to monitor expression by emission of bioluminescence, it was not their purpose to present more details about the transformation procedure by electroporation, which were reported elsewhere [34]. However, the levels of expression of the promoters we tested in our transformed lines were very variable, as we showed by testing the expression from 16 independent transformants for each construction (Figure 4). The silencing of transgenes has been extensively documented in the past (see [71,72] for reviews), and copy number can be positively or negatively correlated with gene expression [73,74]. The possible reasons for this variability include insert copy number, position effect of the site of integration, non-coding-RNA-mediated silencing, methylation of transforming DNA and chromatin conformation at the integration site. Since our main objectives here were to improve transformation frequency and to identify promoters that would be of use for future use in host–virus interaction studies, we did not pursue a more extensive analysis about this variability, which could be a subject of further research, particularly since certain key genes involved in classical gene silencing by siRNA have not yet been identified in *O. tauri* [75]. The question of the stability of the expression of transformed lines is also a subject that merits future investigation. We did keep some of the transformed lines expressing luciferase from either histone 2A or from α-tubulin promoters in culture, and recently remeasured their bioluminescence after about 400 cell divisions in the absence of genetic selection (no G418, >13 months in culture). This confirmed that all lines retained were expressing luciferase (five lines from each of the constructions) with a relative fluorescence for α-tubulin of 2.9 × 10^3^ ± 2.9 × 10^3^ (26-fold control level) and for histone 2A, 7.8 × 10^3^ ± 3.7 × 10^3^ (75-fold control level). We have no reason to believe that the stabilities of PEG-derived transformants would differ from those of electroporation-derived transformants.

Whereas the stress of prasinovirus infection almost always induces rearrangements in the size of chromosome 19 in *O. tauri* [76,77], no changes in the karyotype were visible following PEG-mediated transformation (Figure 4), indicating that the stress of the transformation treatment did not result in chromosomal rearrangements. Our protocol is thus suitable for investigating genes involved in host–virus interactions independently of this response, and will facilitate approaches that require an efficient delivery of transforming DNA, such as homologous (targeted) recombination [15].

## 5. Conclusions

Several different aspects of the *O. tauri* transformation procedure have been improved in this work: (i) the frequency of transformation was increased by 20–40-fold; (ii) the procedure was simplified by the use of PEG, which renders centrifugation and resuspension of cells in a non-ionic solution for electroporation unnecessary; (iii) the procedure was shortened, so that it could be completed in one instead of two days; and (iv) accuracy in determining the transformation frequency was improved. These ameliorations will widen the accessibility of the technique to more plant cell culture laboratories and facilitate its use for applications requiring high frequencies of independent transformants, such as the mutagenesis of dispensable genes involved in responses to viral infection [76,77].

## Supplementary Materials

The following are available online at https://www.mdpi.com/2073-4425/10/5/399/s1, Table S1: Nucleotide sequences of the vector component parts used for transformations; Table S2: Optimization of PEG transformation conditions; Table S3: Raw bioluminescence data; Figure S1: Flow cytometric estimation of viable cells during treatment with PEG, estimated by autofluorescence of chlorophyll.
