# Supplementary material for: Simplified Transformation of Ostreococcus tauri Using Polyethylene Glycol
Source: Genes (Basel). 2019 May 26;10(5):399. doi: 10.3390/genes10050399 (PMC6562926; doi:10.3390/genes10050399)
Supplement: Supplementary file 1 [file genes-10-00399-s001.zip › Additional_file_3_Sanchez_et_al_2nd_revision_Supplementary_genes-475097_FigS1A-B_edited_20190514.pdf]

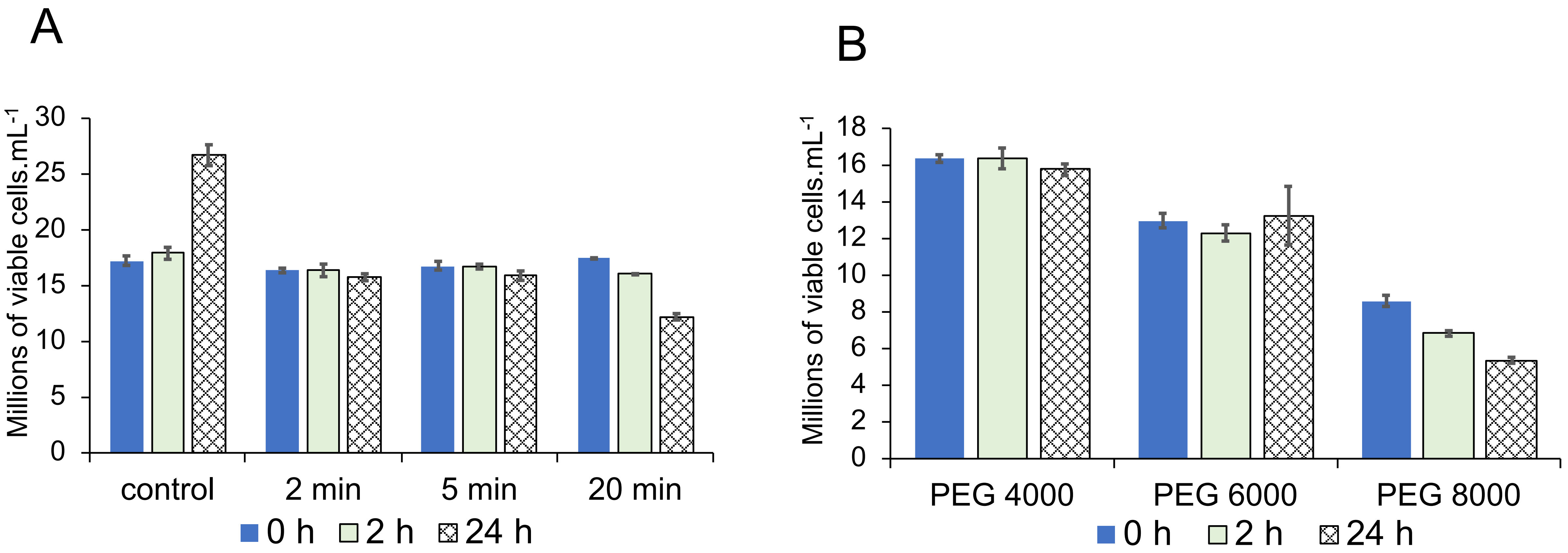

S1A and S1B. Flow cytometric estimation of viable cells after treatment with the PEG DNA mix, estimated by autofluorescence of chlorophyll. Each column error bar shows the standard deviations for 3 independent tests.

Left: control with no PEG, then after 0, 2, 5 or 20 min in the PEG MW 4000/DNA transformation mix. Cytometric measurements were done immediately after treatment (blue bars) then after 2 h (light green), and after 24 h (hatched pattern)

Right: The effects of different MW PEG on cell survival. All PEG/DNA/cell transformation mixes were done for 2 min.

Measurements were made after different recovery times in the culture room following the treatment, (T0, blue), 2 h after the treatment (T2, light green) and 24 h after the treatment (T24, hatched pattern). The 0 h cells were not treated with PEG.
